# Low Prevalence of Cardiomyopathy in Patients with Mitochondrial Disease and Neurological Manifestations

**DOI:** 10.3390/jcdd9070221

**Published:** 2022-07-09

**Authors:** Anish Nikhanj, Jesi Bautista, Zaeem A. Siddiqi, Cecile L. Phan, Gavin Y. Oudit

**Affiliations:** 1 Division of Cardiology, Department of Medicine, Faculty of Medicine and Dentistry, University of Alberta, Edmonton, AB T6G 2R3, Canada; anikhanj@ualberta.ca; 2 Mazankowski Alberta Heart Institute, University of Alberta, Edmonton, AB T6G 2R3, Canada; 3 Division of Neurology, Department of Medicine, Faculty of Medicine and Dentistry, University of Alberta, Edmonton, AB T6G 2R3, Canada; jesi.bautista@gmail.com (J.B.); zsiddiqi@ualberta.ca (Z.A.S.); phan@ualberta.ca (C.L.P.)

**Keywords:** mitochondrial disease, cardiomyopathy, heterogeneity, cardiac imaging

## Abstract

Patients with mitochondrial diseases can develop cardiomyopathy but with variable expressivity and penetrance. Our prospective study enrolled and evaluated a cohort of 53 patients diagnosed with chronic progressive ophthalmoplegia (CPEO, *n* = 34), Kearns–Sayre syndrome (KSS, *n* = 3), neuropathy ataxia and retinitis pigmentosa (NARP, *n* = 1), myoclonic epilepsy with ragged red fibers (MERRF, *n* = 1), Harel–Yoon Syndrome (HYS, *n* = 1) and 13 patients with undefined mitochondrial diseases, presenting primarily with neurological symptoms. Over a 4-year period, six patients in our study cohort were diagnosed with heart disease (11.3%), with only three patients having defined cardiomyopathy (5.7%). Cardiomyopathy was present in a 21-year-old patient with HYS and two CPEO patients having mild cardiomyopathy at an older age. Two CPEO patients had congenital heart disease, and a third CPEO had LV hypertrophy secondary to hypertension. In three patients, traditional risk factors for heart disease, including dyslipidemia, hypertension, and respiratory disease, were present. The majority of our adult cohort of patients have normal cardiac investigations with a median left ventricular (LV) ejection fraction of 59.0%, indexed LV mass of 67.0 g/m^2^, and normal diastolic and valvular function at baseline. A 12-lead electrocardiogram showed normal cardiac conduction across the study cohort. Importantly, follow-up assessments showed consistent cardiac structure and function. Our study shows a low prevalence of cardiomyopathy and highlights the breadth of phenotypic variability in patients with mitochondrial disorders. The presence of cardiovascular risk factors and aging are important comorbidities in our cohort.

## 1. Introduction

Mitochondrial diseases are a group of disorders caused by mitochondrial deoxyribonucleic acid (mtDNA) or nuclear DNA mutations that ultimately lead to defective oxidative phosphorylation [1,2,3]. They may be inherited maternally through mtDNA, through autosomal dominant or recessive patterns, often presenting as a congenital disease [4]. A cardinal feature of mitochondrial diseases is the intrinsic variability of phenotype due to heteroplasmy and the threshold effect [5,6,7,8,9,10,11,12,13,14,15]. Even in the absence of clinical heart disease, accumulation of mutated forms of mtDNA occurs in association with advancing age and delayed presentation [16]. In patients with neurological disease linked to mtDNA mutations, common neuromuscular signs and symptoms include ptosis and ophthalmoplegia, limb weakness, myalgia, exercise intolerance, and fatigue [4,17]. The prevalence of heart disease remains highly variable in this patient population [7,18]. Our study investigated a cohort of patients with chronic progressive external ophthalmoplegia (CPEO), Kearns–Sayre syndrome (KSS), neuropathy ataxia and retinitis pigmentosa (NARP), myoclonic epilepsy with ragged red fibers (MERRF), Harel–Yoon Syndrome, and a group of patients with undefined mitochondrial disease, to determine the prevalence and type of cardiomyopathy in a cohort presenting primarily with neurological manifestations.

## 2. Methods

A cohort of 53 patients with mitochondrial diseases was seen at the Neuromuscular Multidisciplinary (NMMD) clinic at the Kaye Edmonton Clinic (Edmonton, AB, Canada), where they received multifaceted care from specialized physicians in cardiology, neurology, pulmonary medicine, and physiatry in conjunction with allied health care professionals as part of a collaborative multidisciplinary care pathway. Diagnoses included CPEO (34 patients), KSS (3 patients), NARP (1 patient), MERRF (1 patient), Harel–Yoon Syndrome (1 patient), or an undefined mitochondrial disease (13 patients), determined through genetic testing and skeletal muscle biopsy. Patients provided informed written consent before their enrolment, and our study was concordant with ethical guidelines established by the Health Research Ethics Board at the University of Alberta. 

Patient clinical profiles were created and comprised of demographic data, clinical history, including medical and device intervention, and biochemical testing results obtained by electronic chart review. Dyslipidemia was defined in accordance with the 2016 Canadian Cardiovascular Society guidelines [19], and hypertension was defined in accordance with the 2017 American College of Cardiology guidelines [20]. Respiratory disease was defined as chronic obstructive pulmonary disease, asthma, recurrent aspiration pneumonia, respiratory muscle weakness, or restrictive lung disease. Motor and sensory signs and symptoms were documented, and cognitive ability was assessed by the Montreal Cognitive Assessment Score [21]. Cardiac assessments, including 12-lead electrocardiogram (ECG), Holter monitor, transthoracic echocardiogram (TTE), and cardiac magnetic resonance (CMR), were also recorded at baseline and assessed relative to their respective clinical guidelines [22,23,24]. Our investigation also obtained follow-up 12-lead ECG data for 31 patients over a median follow-up period of 2.64 years and follow-up cardiac imaging for 23 patients over a median follow-up period of 3.18 years for patients at greater risk for heart disease on the basis of risk factors and comorbidities. All continuous variables associated with serial cardiac assessment were analyzed using a Mann-Whitney U test. All statistical analyses were performed in R version 4.0.3, and a *p*-value < 0.05 was considered significant.

## 3. Results

**Figure 1:**
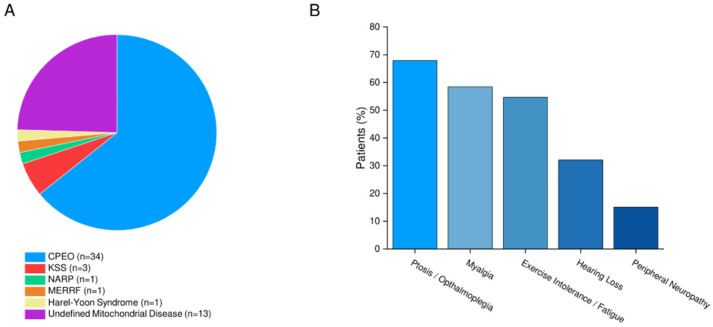
Specific diagnoses of patients in our mitochondrial disease cohort (**A**) and the most prevalent motor and sensory signs and symptoms (**B**). CPEO = chronic progressive ophthalmoplegia; KSS = Kearns–Sayre syndrome; NARP = neuropathy ataxia and retinitis pigmentosa; MERRF = myoclonic epilepsy with ragged red fibers.

Our mixed mitochondrial disease cohort of 53 patients was comprised of 19 (35.8%) men and 34 (64.2%) women with a median age of 50.0 (IQR 33.0 to 61.0) years based on genetic testing and skeletal muscle biopsy (Figure 1A, Table 1). There was a high prevalence of comorbidities, including diabetes, dyslipidemia, and hypertension in these patients (Table 1). Furthermore, there was a high prevalence of respiratory disease (20 (37.7%) patients) and SDOB (11 (20.8%) patients), which was managed by lung volume recruitment and non-invasive ventilation, respectively. Seventeen (32.1%) patients are current smokers or had a history of smoking. Common muscle and sensory abnormalities in this cohort included ptosis or ophthalmoplegia in 36 (67.9%) patients, myalgias in 31 (58.5%) patients, reported exercise intolerance or fatigue in 29 (54.7%) patients, hearing loss in 17 (32.1%) patients, and peripheral neuropathy in eight (15.1%) patients (Figure 1B). Other neurological abnormalities included retinitis pigmentosa in six (11.3%) patients, cataracts in five (9.43%) patients, and cognitive impairment and migraines or headaches in four (7.55%) patients. Mobility aids were only used for five patients (9.43%). Plasma biomarker levels were unremarkable, though creatine kinase levels were noted to be mildly elevated (median level of 99.0 (IQR 72.8 to 218.5) U/L; Table 1). Importantly for the broader cohort, B-type natriuretic peptide (BNP) levels were normal, with a median of 14.0 (IQR 7.00 to 37.0) pg/mL.

At the time of NMMD clinic enrollment, all patients received a 12-lead ECG, which showed normal sinus rhythm with preserved cardiac conduction; one patient (1.89%) had a left anterior fascicular block (Table 2). Further, 24- and 48-hHolter monitoring was performed in 19 patients with no detected brady-arrhythmias or tachy-arrhythmias except in one patient where atrial fibrillation was detected. Forty patients received a TTE, while thirteen patients received a cardiac MRI. Echocardiographic analysis showed a median left ventricular internal dimension at end-diastole (LVIDd) of 4.30 (IQR 4.10 to 4.70) cm, a median left ventricular internal dimension at end-systole (LVIDs) of 2.80 (IQR 2.52 to 3.03) cm, and a median left ventricular mass index (LVMI) of 67.0 (IQR 56.1 to 76.3) g/m^2^. Left ventricular ejection fraction (LVEF) was 59.0 (IQR 58.0 to 61.8) %. Left atrial volume and diastolic function were normal (Table 2). Assessment of right ventricular (RV) size and function was also unremarkable. Furthermore, TTE showed no overt valvular disease in these patients. Cardiac magnetic resonance imaging in the other 13 patients revealed similar findings to those obtained from TTE, namely normal LV volumes and mass and systolic function. The LV end-diastolic volume index was 66.0 (IQR 54.5 to 74.5) mL/m^2^, LV end-systolic volume index was 27.0 (IQR 25.2 to 28.0) mL/m^2^, and the LVMI was 44.0 (IQR 34.0 to 51.0) g/m^2^. Additionally, the median LVEF was 62.0 (IQR 56.0 to 64.0) %. Left atrial volume, as well as RV volumes and systolic function (median RVEF of 55.0 (IQR 52.0 to 58.0) %), were normal (Table 2). None of the patients who received CMR imaging showed evidence of myocardial fibrosis when screened with gadolinium contrast. 

**Figure 2:**
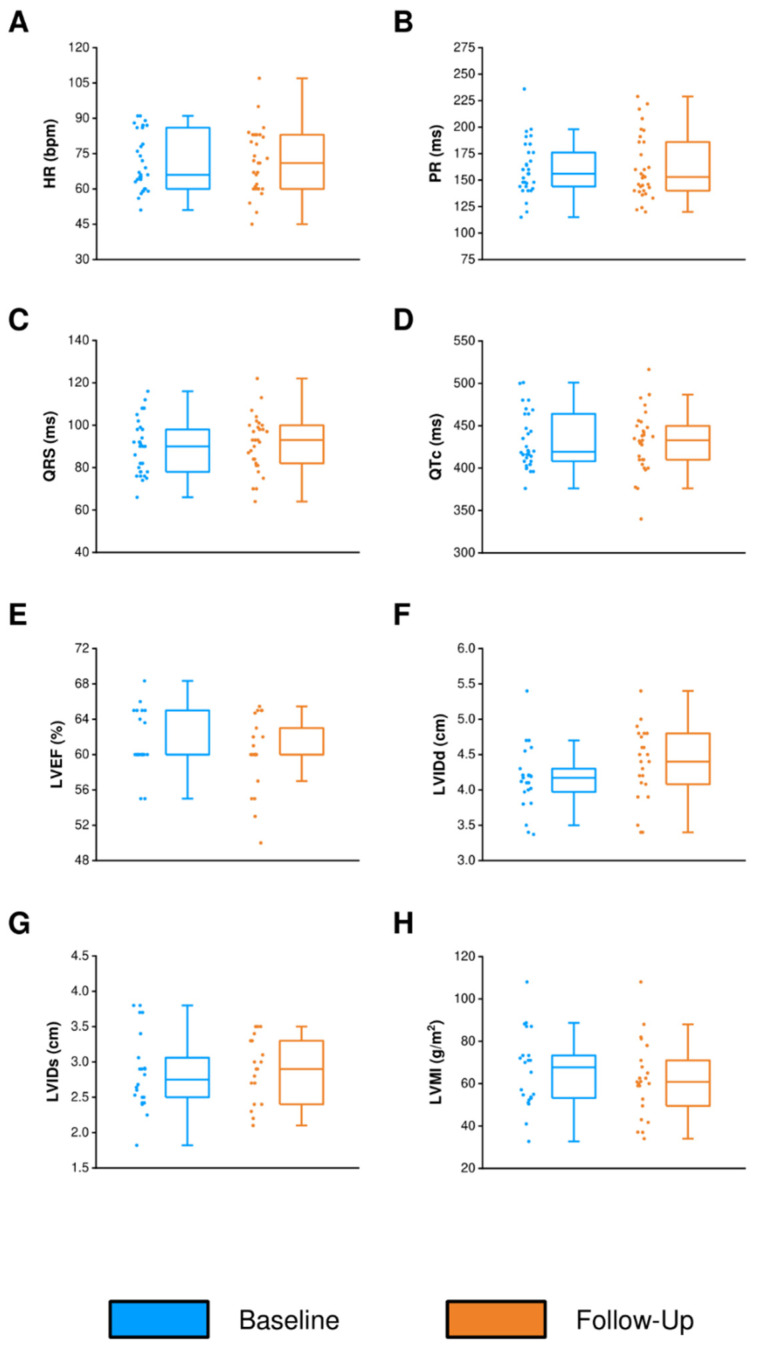
Serial 12-lead electrocardiogram assessment (*n* = 31) (**A**–**D**) and serial cardiac imaging assessment by transthoracic echocardiogram (*n* = 19) and cardiac magnetic resonance (*n* = 4) (**E**–**H**). HR = heart rate; LVEF = left ventricular ejection fraction; LVIDd = left ventricular internal dimension at end diastole; LVIDs = left ventricular internal dimension at end systole; LVMI = left ventricular mass index.

We obtained follow-up data in a subset of our patients in which serial 12-lead ECG or imaging was clinically indicated. Follow-up 12-lead ECG data for 31 patients over a median period of 2.64 (IQR 1.77 to 3.73) years showed sinus rhythm and no significant conduction abnormalities, and conduction parameters remained stable (Figure 2B–D). Patient PR intervals were consistent from 156.0 (IQR 144.0 to 180.0) ms to 153.0 (IQR 140.0 to 186.0) ms over the follow-up period (*p* = 0.67; Figure 2B). Similarly, QRS durations were consistent from 90.0 (IQR 78.0 to 98.0) ms to 93.0 (IQR 83.0 to 99.5) ms over the follow-up period (*p* = 0.40; Figure 2C). We also collected follow-up TTE data for 19 patients and CMR data for four patients over a median period of 3.18 (IQR 2.72 to 3.99) years (Figure 2E–H). Patient LV systolic function was maintained and LVEF was maintained at 60.0 (IQR 59.0 to 62.7)% to 60.0 (IQR 57.8 to 60.8)% over the follow-up period (*p* = 0.67; Figure 2E). Furthermore, patient LVIDd was consistent from 4.11 (IQR 3.98 to 4.21) cm to 4.25 (IQR 3.93 to 4.79) cm (*p* = 0.09; Figure 2F), patient LVIDs was consistent from 2.75 (IQR 2.50 to 3.02) cm to 2.90 (IQR 2.55 to 3.30) cm (*p* = 0.71; Figure 2G) and LVMI was consistent from 67.7 (IQR 53.3 to 73.3) g/m^2^ to 60.8 (IQR 51.1 to 69.5) g/m^2^ (*p* = 0.45; Figure 2H) over the follow-up period.

There were six patients in our cohort that were diagnosed with heart disease (Table 3). Patient 1, a 21-year-old patient with Harel–Yoon Syndrome with a clinical history of neurological morbidities, was seen at the NMMD clinic, and a 12-lead ECG showed sinus tachycardia and normal cardiac conduction. Transthoracic echocardiography showed moderate left ventricular dysfunction (LVEF of 38.0%) with normal LV structure and a small left atrium (LA). Right ventricular structure and systolic function were preserved, and there were no indications of valvular disease. The patient had their angiotensin-converting enzyme inhibitor (ACEi) and beta-blocker uptitrated. 

Patient 2, a 61-year-old female with CPEO and comorbidities including dyslipidemia and respiratory disease, had a normal 12-lead ECG and TTE showed mild LV systolic, and diastolic dysfunction with normal valves, and the patient had an unremarkable angiogram. The patient was prescribed both an ACEi and beta-blocker. Patient 3, a 78-year-old male with CPEO, respiratory disease as a comorbidity, and a clinical history of symptomatic non-sustained VT (syncope), had a 12-lead ECG that showed normal sinus rhythm, first-degree atrioventricular block, and two premature ventricular contractions. Transthoracic echocardiography showed a mild reduction in LV systolic function (LVEF of 45.0%), LV hypertrophy (LVMI of 108.0 g/m^2^), and LA dilation (LAVI of 40.6 mL/m^2^). Importantly, a diagnostic coronary angiogram was normal. The patient was prescribed an ACEi, had their beta-blocker uptitrated, and received a dual-chamber implantable cardiac defibrillator.

Patient 4 was a 26-year-old female with CPEO and a family history of congenital heart disease. A 12-lead ECG showed RV hypertrophy, and a TTE study showed RV dilation and hypertrophy with preserved valve function. A transesophageal echocardiogram revealed secundum atrial septal defect (ASD) with significant left-to-right shunting, and she is scheduled for percutaneous closure of her ASD. Patient 5, a 59-year-old male with CPEO and an underlying diagnosis of Wolff–Parkinson–White syndrome, had a clinical history of transient ischemic attack, angina, and was a smoker. The patient underwent a successful ablation of his accessory pathway at 35 years of age. The patient developed paroxysmal atrial fibrillation (AF), which was captured by Holter monitoring and a 12-lead ECG. Cardiac imaging by TTE was normal. He responded well to cardioversion, and anticoagulation was initiated (Table 3). Patient 6, a 52-year-old male with a clinical history of neurological morbidities and syncope, presented with hypertension (systolic blood pressure 144 mmHg, diastolic blood pressure 83 mmHg). A 12-lead ECG showed LV hypertrophy confirmed by TTE (LVMI of 115.5 g/m^2^) associated with LA dilation (left atrial volume index (LAVI) of 45.0 mL/m^2^). The patient’s ACEi uptitrated, and with dietary changes and improved adherence, his hypertension was well controlled. 

## 4. Discussion

Mitochondrial disorders would conceivably have the most prominent manifestations of disease in tissues with high-energy demand. This is of particular relevance to the heart and cardiomyocytes, which have the highest volume density of mitochondria, and, therefore, the potential for cardiac involvement must be recognized [25,26]. Phenotypes of hypertrophic cardiomyopathy (HCM) and dilated cardiomyopathy (DCM) resulting in heart failure and arrhythmias, including cardiac conduction abnormalities, can occur in patients with mitochondrial disorders [9,10,11,12,13,14,15]. However, given the marked phenotypic variability seen in mitochondrial disorders [5,6,7,8,9,10,11,12,13,14,15], characterizing the type and degree of heart disease in patients with mtDNA mutations remains an important task. In our study cohort, heart disease was present in six patients, and cardiomyopathy secondary to mitochondrial disease was diagnosed in three of these patients (5.7%). Our findings are consistent with a recent report on cardiac manifestations in adult mitochondrial disease caused by nuclear genetic defects, where the directly attributable prevalence was only 4.8%, with a significant burden of acquired heart disease due to conventional cardiovascular risk factors [27].

Our patient with Harel–Yoon syndrome presented at 21 years old with no risk factors for heart disease, and, therefore, the cardiomyopathy was secondary to the underlying mitochondrial disease. Two patients with CPEO (61 and 78 years old) had reduced systolic function and structural heart disease and underlying risk factors, including respiratory disease and dyslipidemia, and were of older age. The accumulation of mutated forms of mtDNA with aging can contribute to the delayed presentation of the mild cardiomyopathy seen in these two patients [16]. With regards to the broader cohort, there was a high prevalence of diabetes, dyslipidemia, hypertension, and respiratory disease, which are risk factors for heart disease and major adverse cardiac events [22,28]. Furthermore, these are prevalent in the setting of sedentary lifestyles in this patient population, and, therefore, ongoing monitoring is justified [29]. It is important to consider underlying risk factors for heart disease in these patients in the same light as their diagnosis of mitochondrial disease. These factors are relevant when considering the 52-year-old CPEO patient with LV hypertrophy secondary to hypertension. Additionally, two patients with CPEO were diagnosed with CHD, namely, secundum ASD and Wolff–Parkinson–White syndrome. Symptoms of heart disease observed in both patients were characteristic of the underlying CHD, including RV dilation and hypertrophy, and atrial fibrillation, respectively, and therefore not attributed to the coexisting diagnosis of mitochondrial disease.

Our study showed a markedly low prevalence of cardiomyopathy in patients with CPEO, KSS, NARP, and MERRF, which was supported by follow-up data that supported our baseline observations and showed no indications of disease progression from a cardiac perspective. As patients age, a diagnosis of heart disease can likely be attributed to risk factors as opposed to an underlying diagnosis of mitochondrial disease. Traditional risk factors should be recognized to ensure efficient and effective care is provided with appropriate medical and device intervention when appropriate. Moreover, we recognize the impact of mitochondrial diseases on cardiac outcomes and believe our findings reflect the phenotypic variability of these diseases, which justifies active monitoring in these patients. In addition, given the documented prevalence of cardiac conduction abnormalities in patients with mitochondrial myopathies [12,13,30], 12-lead ECG studies remain a necessary means of assessing cardiac electrophysiology, and we have demonstrated its utility in patients with muscular dystrophy (MD) [31]. Transthoracic echocardiography remains a common and feasible method for assessing cardiac structure and function in patients with neuromuscular diseases, given the burden of comorbidities. Additionally, the use of cardiac biomarkers, including high-sensitive troponin assays, is a minimally invasive and feasible modality for cardiac assessment with predictive value [32,33].

We recognize the limitations of this study. Given the rarity of the mitochondrial diseases we have studied, we were limited in the ability to perform subgroup analysis. This encourages further consenting of patients to create a more comprehensive investigation. Patients with these specific mitochondrial diseases share similar signs of muscle weakness and fatigue as other inherited neurological disorders [10,34,35], though they differ in their absence of heart disease. In conclusion, these findings serve to inform clinicians of our findings, which showed an absence of cardiomyopathy secondary to mitochondrial disease in this patient cohort. A composite of our findings with previous research demonstrates the variability of cardiac involvement in these patients and therefore encourages further disease surveillance

## Figures and Tables

**Table 1:** Clinical characteristics of our cohort with mitochondrial diseases.

Variable	Mitochondrial Disease (*n* = 53)
**Men/Women, No.**	19 (35.8%)/34 (64.2%)
**Age, Yrs.**	50.0 (33.0–61.0)
**Height, cm**	165.1 (160.0–175.4)
**Weight, kg**	73.0 (59.8–82.0)
**Body Surface Area, m^2^**	1.80 (1.60–2.03)
**Current/Former Smoker, No.**	17 (32.1%)
**Ambulatory Aids, No.**	5 (9.43%)
**Comorbidities, No.**	
Diabetes	5 (9.43%)
Dyslipidemia *	9 (17.0%)
Hypertension ^†^	7 (13.2%)
Respiratory Disease	20 (37.7%)
Sleep Disordered Breathing	11 (20.8%)
**Respiratory Therapies, No**.	
Lung Volume Recruitment	5 (9.43%)
Non-Invasive Ventilation	5 (9.43%)
**Vitals, median**	
Heart Rate, bpm	80.0 (65.0–87.5)
Systolic Blood Pressure, mmHg	125.0 (116.5–135.5)
Diastolic Blood Pressure, mmHg	75.0 (66.5–82.0)
**Biomarkers, median**	
B-Type Natriuretic Peptide	14.0 (7.00–37.0)
Creatine Kinase	99.0 (72.8–218.5)
Creatinine	63.0 (50.0–79.5)
C-Reactive Protein	3.00 (1.40–8.80)
Potassium	4.40 (4.10–4.70)

* Dyslipidemia defined as low-density lipoprotein cholesterol ≥3.5 mmol/L or non-high-density lipoprotein cholesterol ≥4.3 mmol/L. ^†^ Hypertension defined as systolic blood pressure >130 mmHg or diastolic blood pressure >80 mmHg.

**Table 2:** Baseline cardiac assessment of our cohort with mitochondrial diseases.

Modality	Mitochondrial Disease
**12-Lead Electrocardiogram**	**(*n* = 53)**
Heart Rate, bpm	71.0 (62.0–82.5)
PR Interval, ms	155.5 (144.0–171.3)
QRS Duration, ms	90.0 (79.0–98.0)
QT Interval, ms	396.0 (372.0–415.5)
QTc Bazett, ms	425.5 (414.3–443.0)
Advanced Atrioventricular Block	0
Left Anterior Fascicular Block	1 (1.89%)
**Echocardiogram**	**(*n* = 40)**
Left Atrial Volume Index (mL/m^2^)	20.2 (16.5–25.1)
Left Ventricular Internal Dimension at End-Diastole (cm)	4.30 (4.10–4.70)
Left Ventricular Internal Dimension at End-Systole (cm)	2.80 (2.52-3.03)
Left Ventricular Posterior Wall Thickness at End-Diastole (cm)	0.88 (0.77–0.99)
Left Ventricular Ejection Fraction (%)	59.0 (58.0–61.8)
Left Ventricular Mass Index (g/m^2^)	67.0 (56.1–76.3)
E/e’	7.55 (6.40–8.93)
Mitral Valve E/A	1.30 (0.99–1.47)
Mitral Valve Deceleration Time (ms)	186.0 (168.0–262.0)
Tricuspid Annular Plane Systolic Excursion (mm)	2.24 (1.98–2.53)
Right Ventricular Systolic Pressure (mmHg)	25.0 (23.3–26.1)
Right Ventricle Size	Normal
Right Ventricular Systolic Function	Normal
**Cardiac Magnetic Resonance Imaging**	**(*n* = 13)**
Left Atrial Volume Index (mL/m^2^)	27.0 (22.0–33.4)
Left Ventricular End Diastolic Volume Index (mL/m^2^)	66.0 (54.5–74.5)
Left Ventricular End Systolic Volume Index (mL/m^2^)	27.0 (25.5–28.0)
Left Ventricular Ejection Fraction (%)	62.0 (56.0–64.0)
Left Ventricular Mass Index (g/m^2^)	44.0 (34.0–51.0)
Right Ventricular End Diastolic Volume Index (mL/m^2^)	70.0 (60.5–79.5)
Right Ventricular End Systolic Volume Index (mL/m^2^)	34.0 (26.0–38.0)
Right Ventricular Ejection Fraction (%)	55.0 (52.0–58.0)

**Table 3:** Clinical characteristics of patients with mitochondrial disease with heart disease at baseline visit (*n* = 6).

Patient/Diagnosis	Age (yrs)/Sex	Clinical History and Comorbidities	Cardiac Abnormality	HR (bpm)	sBP (mmHg)/dBP (mmHg)	ECG Findings (ms)	TTE Findings	Before Assessment	After Assessment	
								Cardiac Medication and Daily Dose (mg)	Cardiac Medication and Daily dose (mg)	Cardiac Device
1/Harel–Yoon Syndrome	21/M	Spastic paraplegia (spasticity, weakness, ataxia), neuropathy, seizures	Moderate LV systolic dysfunction	133	117/75	PR: 131; QRS: 80 QTc: 421 Sinus tachycardia	LVEF: 38.0% LVMI: 50.7 g/m^2^ LVIDd: 3.90 cm LVIDs: 3.40 cm LAVI: 9.00 mL/m^2^	Perindopril 2 mg, Bisoprolol 1.25 mg	Perindopril 4 mg, Bisoprolol 2.5 mg	NA
2/CPEO	61/F	Dyslipidemia, respiratory disease (exertional dyspnea, SDOB)	Mild LV systolic and diastolic dysfunction	65	117/70	PR: 160; QRS: 105 QTc: 449	LVEF: 53.0% LVMI: 78.0 g/m^2^ LVIDd: 4.80 cm LVIDs: 3.50 cm LAVI: 27.0 mL/m^2^	NA	Perindopril 2 mg, Bisoprolol 2.5 mg	NA
3/CPEO	78/M	Syncope, respiratory disease (oropharyngeal dysphagia)	Mild LV systolic dysfunction, Non-sustained VT	60	124/72	PR: 232; QRS: 108 QTc: 456 1st degree AVB, 2 PVCs	LVEF: 45.0% LVMI: 108.0 g/m^2^ LVIDd: 5.40 cm LVIDs: 3.40 cm LAVI: 40.6 mL/m^2^	Metoprolol 25 mg	Perindopril 4 mg, Metoprolol 100 mg	Dual-chamber ICD
4/CPEO	26/F	Severe fatigue, migraines, myalgia	Secundum ASD, RV dilation, RV hypertrophy	70	118/80	PR: 164; QRS: 89 QTc: 374	LVEF: 60.0% LVMI: 49.6 g/m^2^ LVIDd: 3.40 cm LVIDs: 2.60 cm LAVI: 15.0 mL/m^2^	NA	NA	NA
5/CPEO	59/M	Smoker, TIA Angina	Atrial Fibrillation (Wolff–Parkinson–White Syndrome)	103	90/62	PR: 155; QRS: 95 QTc: 377	LVEF: 55.0% LVMI: 66.2 g/m^2^ LVIDd: 4.50 cm LVIDs: 3.30 cm LAVI: 16.8 mL/m^2^	NA	Apixaban 10 mg	NA
6/CPEO	52/M	Syncope, falls, dysautonomia, neuropathy, seizures	LV hypertrophy, LA enlargement	71	144/83	PR: 176; QRS: 106 QTc: 513 LV hypertrophy, P mitrale	LVEF: 60.0% LVMI: 115.5 g/m^2^ LVIDd: 4.40 cm LVIDs: 3.30 cm LAVI: 45.0 mL/m^2^	Perindopril 4 mg	Perindopril 8 mg	NA

AF = atrial fibrillation; ASD = atrial septal defect; AVB = atrioventricular conduction block; CPEO = chronic progressive external opthalmoplegia; dBP = diastolic blood pressure; ECG = electrocardiogram; HR = heart rate; ICD = implantable cardiac defibrillator; LA = left atrial; LV = left ventricular; LAVI = left atrial volume indexed; LVEF = left ventricular ejection fraction; LVIDd = left ventricular internal dimension at end diastole; LVIDs = left ventricular internal dimension at end-systole; LVMI = left ventricular mass indexed; NA = not applicable; PVC = premature ventricular contraction; RV = right ventricular; sBP = systolic blood pressure; SDOB = sleep disordered breathing; TIA = transient ischemic attack; TTE = transthoracic echocardiogram; VT = ventricular tachycardia.

## Data Availability

The data presented in this study are available on request from the corresponding author.
